# C-Reactive Protein Levels in Children with Acute Bronchiolitis

**DOI:** 10.1155/2022/1311936

**Published:** 2022-05-23

**Authors:** Hasan M. Isa, Abdulrahman D. Mohroofi, Fatema N. Alkhan, Asma Z. Hasan, Mariam M. Alkubisi, Sana S. Alhewaizem, Sara I. Khalifa, Noora G. Alromaihi

**Affiliations:** ^1^Consultant Pediatric Gastroenterologist, Pediatric Department, Salmaniya Medical Complex, Arabian Gulf University, Manama, Bahrain; ^2^Senior House Officer, Pediatric Department, King Hamad University Hospital, Muharraq, Bahrain; ^3^Salmaniya Medical Complex, Manama, Bahrain; ^4^Sulwan Psychiatric Hospital, Bu Quwah, Bahrain; ^5^Obstetrics and Gynecology Department, King Hamad University Hospital, Muharraq, Bahrain; ^6^Dream Reem Medical Center, Muharraq, Bahrain; ^7^Pathology Department, Bahrain Defense Force Hospital, Riffa, Bahrain

## Abstract

**Results:**

Of 287 patients, 229 (79.2%) were included. 132 (57.6%) were males. Median presentation age was 3.7 (interquartile range (IQR), 1.27-12.33) months. Median CRP level was 10.4 (IQR, 2.8-35.1) mg/L. CRP was high in 167 (72.9%) patients. 17.6% (33/187 patients) had confirmed bacterial coinfection. Respiratory syncytial virus (RSV) was detected in 84 (36.7%) patients. Mean CRP level was higher in RSV-negative compared to RSV-positive patients, 31.3 ± 44.3 versus 21.5 ± 27.7 mg/L, respectively (*P* = 0.042). Respiratory viral serology profile was positive in 34.7% (17/49 patients). 66.9% (107/160 patients) had positive chest X-ray. Antibiotics were used in 78.1% (179/227 patients). Thirteen (5.7%) patients required intensive care, five (2.2%) had surgical intervention, four (1.8%) required endotracheal intubation, and four (1.8%) died. Patients with high CRP were older at presentation (*P* < 0.0001) and had more fever (*P* < 0.0001) and cough (*P* = 0.002), but lower hemoglobin level (*P* < 0.0001) compared to those with normal CRP. Fever (*P* = 0.016) and hemoglobin level (*P* = 0.002) were independent factors.

**Conclusion:**

Most children with acute bronchiolitis had high rate of elevated CRP values that did not correlate with the rate of bacterial coinfection. High CRP levels were found in older children, those presented with more fever and cough, and had a lower hemoglobin level despite that those factors were previously reported to be associated with disease severity and bacterial coinfection. This study also showed a high overall rate of antibiotic prescriptions in mostly viral disease.

## 1. Introduction

Acute bronchiolitis is one of the most common respiratory diseases in children younger than two years of age [1]. In most cases, respiratory syncytial virus (RSV) is the cause [1]. By the age of two, nearly all children are infected at least once by RSV bronchiolitis [2]. It is more common in preterm newborns and in male patients [3, 4]. Moreover, it is the leading cause for more than 125,000 hospitalizations and 250 deaths yearly in the United States [1]. Acute bronchiolitis-related morbidity and mortality are much higher in premature infants and in infants with chronic lung disease or congenital heart diseases [2].

C-reactive protein (CRP), which is an acute phase reactant and one of the indicators of acute inflammation, has been linked to bacterial coinfections like bacterial pneumonia [5, 6]. However, it was shown that patients with RSV bronchiolitis, bronchopneumonia, and RSV pneumonia had elevated levels of CRP along with higher white blood cells (WBC) count and erythrocyte sedimentation rate (ESR) which all indicate bacterial coinfection [5–7]. Accordingly, identification of CRP levels can be an important indirect marker for viral infections and an indicator for progression of infection and effectiveness of the treatment [5]. In patients with RSV bronchiolitis, it is worth mentioning that elevated CRP levels were associated with prolonged length of hospital stay [1, 5, 8].

Data about the association between acute bronchiolitis and CRP levels are scares. There are few reports tackling this issue came from Arabian Gulf region and worldwide [5, 9, 10]. In Bahrain, no studies have been published regarding CRP levels in children with acute bronchiolitis. This study is aimed at assessing the frequency of elevated C-reactive protein (CRP) levels in hospitalized children presented with acute bronchiolitis and at comparing the clinical characteristics, laboratory and radiological findings, antibiotics use, and outcome according to the CRP levels.

## 2. Methods

### 2.1. Study Design and Study Participants

This is a retrospective, cross-sectional, and analytical study where the electronic medical records of all patients with a clinical impression of acute bronchiolitis and were admitted to the pediatric department at Salmaniya Medical Complex (SMC), Bahrain in the period between September 1, 2019, and February 29, 2020, were retrieved.

Children below the age of five years who were admitted with acute bronchiolitis, had a nasopharyngeal swab for RSV infection tested via direct antigen detection and/or polymerase chain reaction (PCR), and CRP level checked were included in this study. Patients were suspected to have acute bronchiolitis based on the criteria published by the American Academy of Pediatrics [11]. The criteria indicate that the diagnosis is based on signs and symptoms suggesting bronchiolitis including rhinorrhea, cough, tachypnea, wheezing, rales, and increased respiratory effort manifested as grunting, nasal flaring, and intercostal and/or subcostal retractions [11]. Radiographic or laboratory investigations should not be routinely used to diagnose acute bronchiolitis [11]. CRP levels were tested using enzyme-linked immunosorbent assay (ELISA) technique and presented as quantitative figures. Normal CRP value was ≤3 mg/L.

### 2.2. Data Collection

Demographic data including sex, nationality, gestational age, age at presentation, clinical presentation, length of stay, and age at the time of study were collected. Results of laboratory investigations including complete blood count, CRP levels, blood culture, urine culture, and cerebrospinal fluid (CSF) culture, and nasopharyngeal swab for RSV direct antigen detection and/or PCR were retrieved. Results of respiratory viral serology profile test (immunoglobulin M and G) for legionella pneumophilia, mycoplasma pneumonia, coxiella burnettii, chlamydia pneumonia, adenovirus, RSV, influenza A and B, and parainfluenza were gathered. Radiological findings on the chest X-ray reported by senior radiologists were documented. Medical therapy including antibiotic use, patient's outcome, and complications were also evaluated.

### 2.3. Ethical Approval

This study was conducted in accordance with the Helsinki declaration and was ethically approved by the Research and Research Ethics Committee for Government hospitals, Salmaniya Medical Complex, Bahrain (IRB number: 155131221). Signed informed consent was taken from each child's parent or legal guardian upon admission.

### 2.4. Statistical Analysis

The data were statistically analyzed using SPSS version 21 software. Demographic data were presented as frequencies and percentages. Normally distributed continuous variables were presented as mean and standard deviation (SD). Median and interquartile range (IQR) were calculated for nonnormally distributed variables. Based on CRP results, patients were divided into two groups, high CRP level (group 1) and normal CRP level (group 2). The two groups were compared in terms of demographic data, clinical presentation (fever and cough), laboratory findings (complete blood count, blood, urine, and CSF cultures, and RSV swab for direct antigen detection and/or PCR, and serology), radiological findings (chest X-ray), antibiotic uses, and the outcomes. Chi-Square Fisher's test was used to compare categorical variables. Student's *T*-test or Mann–Whitney *U*-test was used to compare continuous variables. Variables found to be significant in the univariate analysis and had no multicollinearity using a variation inflation factor > 8 were included in a binary logistic regression to detect the independent factors of high CRP levels. *P* value < 0.05 was considered statistically significant. Confidence interval was set at 95%.

## 3. Results

During the study period, a total of 287 patients were admitted with a clinical presentation of acute bronchiolitis. Fifty-eight (20.1%) patients were excluded due to of unavailability of data of CRP levels. The remaining 229 (79.2%) patients were included in the study. Demographic data of the included patients are shown in Table 1.

One hundred and thirty-two (57.6%) patients were males. One hundred and sixty-eight (73.4%) patients were Bahraini, and 61 (26.6%) were non-Bahraini (seven patients were from India and Yemen each, six from Pakistan while one patient from Indonesia, Jordon, Kenya, Saudi Arabia, Libya, Philippine, Syria each, and 35 patients were from nonspecified other countries).

**Figure 1:**
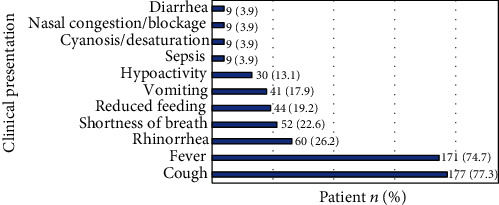
Clinical presentations of 229 patients admitted with acute bronchiolitis.

The most common clinical presentation was cough (177 (77.3%) patients) followed by fever (171 (74.7%) patients) as shown in Figure 1.

Twenty-six (11.4%) patients presented with one or more of other symptoms including congested throat in four patients; jaundice, wheeze, irritability, and convulsions each in two patients; abdominal pain, vomiting, dysphagia, apnea, noisy breathing, hoarseness, stridor, drooling, groaning, decrease micturition, foul smell urine, ear tugging, pallor, and skin rash each in one patient.

Results of the laboratory investigations are shown in Table 2.

One hundred and sixty-six (72.5%) patients were anemic, 114 (49.8%) had leukocytosis, eight (3.5%) had leukopenia, 110 (48%) had thrombocytosis, while eight (3.5%) patients had thrombocytopenia. Median CRP level was 10.4 (IQR, 2.8-35.1) mg/L. CRP level was high in 167 (72.9%) patients while 62 (27.1%) patients had normal level.

Among 187 (81.7%) patients who were tested for bacterial coinfections, 33 (17.6%) patients had confirmed bacterial infection. Seventeen (9.6%) patients had positive blood culture out of 177 (59.2%) tested patients; 13 (11.5%) had positive urine culture out of 113 (43.3%) tested patients; three (10.3%) had positive CSF culture out of 29 (12.7%) patients with lumber puncture.

Out of 229 patients underwent nasopharyngeal swabs for RSV infections, 228 (99.6%) patients were tested via direct antigen detection test, 35 (15.4%) of them were confirmed by PCR, and one (0.4%) patient was tested with PCR only. Median duration between the presentation and the swabbing was 24 (IQR, 0-48) hours. Positive RSV results were found in 84 (36.6%) patients, 142 (62%) had negative results, and three patients' results were still pending at the time of study. The latter were excluded from the analysis of predicted factors for high CRP levels. Mean CRP level was significantly higher in RSV-negative compared to RSV-positive patients, 31.3 ± 44.3 mg/L versus 21.5 ± 27.7 mg/L, respectively (*P* = 0.042, CI = −19.2 to 0.37).

**Figure 2:**
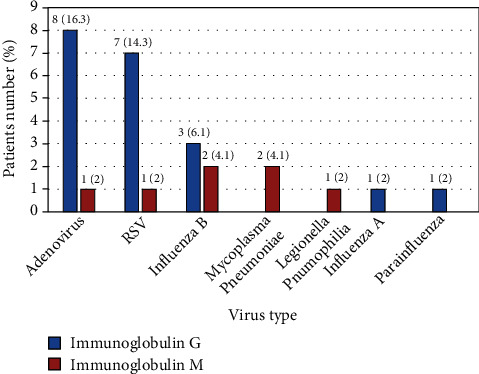
Respiratory viral serology profile of children presented with acute bronchiolitis (17 out 49 tested children were positive, and 10 of them tested positive for more than one virus). RSV: respiratory syncytial virus.

Respiratory viral serology profile was performed in 49 (21.4%) patients. Of them, 17 (34.7%) had positive results (Figure 2), and Coxiella burnettii and chlamydia pneumonia were not identified in any patient.

Chest X-ray data were available for 160 (69.9%) patients; 107 (66.9%) patients had positive findings while the remaining 53 (33.1%) patients had a normal chest X-ray. The radiological findings varied between the patients, and some patients had more than one finding. Hyperinflation was the most common finding and was seen in 48 (44%) patients followed by infiltration of lung fields, bronchovascular marking, opacification, collapse, and blunting of the costophrenic angle (32.7%, 19.6%, 11.2%, 5.6%, and 0.6%, respectively). Other chest X-ray findings were found in 19 (17.8%) patients (congenital pneumonia was found in four patients; white lung was found in three patients; ground glass appearance was found in two patients; bronchopulmonary spasm, unclear cardiac borders, cardiomegaly, lung edema, prominent pulmonary artery, pneumothorax, steeple sign, contusion, narrow mediastinum, and wide mediastinum were found in one patient each).

All the patients were managed by steam inhalation and bronchodilators. Antibiotics were given to 179 (78.1%) out of 227 (99.1%) patients with available data. Hospital outcome was available for 227 (99.1%) patients, 13 (5.7%) patients required admission to the pediatric intensive care unit (PICU), five (2.2%) had a surgical intervention during their admission (tracheostomy in two, while insertion of central line, intercostal drainage tube, and naso-jejunal tube were required in one patient each), four (1.8%) patients required endotracheal intubation, and four patients (1.8%) died. The first patient died was an eight-month-old male ex-preterm who had patent ductus arteriosis, ventricular septal defect, and hydrocephalus. He presented with acute bronchiolitis and sepsis like picture and required intubation and PICU admission. He died after 33 days of hospital stay. The second patient was a 16-day-old male preterm infant who developed collapse consolidation, right pneumothorax, and positive blood culture. He required PICU admission and died after 17 days of hospital stay. The third patient was a one-year-old female ex-preterm with Escobar syndrome (a form of arthrogryposis multiplex congenita) and failure to thrive who presented with pneumonic patch on chest X-ray. She died after 13 days of hospital stay. The fourth patient was a one-year-old female term infant with biventricular hypertrophy who presented with cough and cyanosis. She had a positive RSV swab. She required tracheostomy tube insertion and died after 44 days of hospital stay. Comparison between group 1 (high CRP level) and group 2 (normal CRP level) is shown in Table 3.

Patients with high CRP level were older at presentation (*P* < 0.0001), older at the time of study (*P* < 0.0001), had more frequent fever (*P* < 0.0001), more cough (*P* = 0.002) but lower hemoglobin level (*P* < 0.0001) compared to those with normal CRP level. Despite that, the mean CRP level in the present study was higher in patients admitted to the PICU compared to those not, 34.8 ± 48.2 versus 27.1 ± 38.6, respectively, this difference was not statistically significant (*P* = 0.804). Other variables such as sex, nationality, gestational age, length of stay, white blood cells count, platelets count, and positive blood, urine, and CSF cultures, positive chest X-rays, positive RSV swabs, antibiotic use, complications, mortality, outcome showed no significant difference between the two groups. The significant variables were tested for multicollinearity (VIF > 8) between each other and were put into a logistic regression model. Accordingly, fever (*P* = 0.016) and hemoglobin level (*P* = 0.002) were found to be the independent predictor for high CRP levels (Table 4).

## 4. Discussion

Inflammatory biomarkers such as CRP can aid confirm the clinical suspicion of invasive bacterial infection and optimize and tailor antibiotic therapy [6, 12]. However, elevated serum CRP levels have been witnessed in children with acute bronchiolitis in the absence of a confirmed bacterial coinfection or the need of antibiotic used [13]. In this study, CRP levels were high in 73.3% (170/229) of the patients which is comparable to the percentage reported by Lamarão et al. (77.1%) [14]. Yet, several studies reported lower percentages, ranging from 1.5% to 62.5% [5, 8, 9, 15]. High CRP levels were associated consistently with bacterial infections but inconsistently with viral infections as shown by Peltola et al.'s study [7]. Although high CRP levels were reported to be more associated with bacterial infections, this was not the case in our study as only 9.6% (17/177), 10.3% (3/29), and 11.5% (13/113) of our patients had positive blood, CSF, and urine cultures, respectively.

Confirming the findings of several other studies, this study showed a higher percentage of males (57.6%) presented with acute bronchiolitis compared to females (42.4%) [4, 15–17]. RSV infection predominance in males is well-known but its mechanism has not been explored up till now [16]. This finding might be attributed to the suppression of blood eosinophil cell count or due to the immunosuppressive effect of male hormones [16]. In our study, male patients had higher CRP levels (99/132, 75%) compared to females (68/97, 70.1%). Yet, sex was not a significant risk factor for high CRP. Conversely, Nagayama et al. showed that higher CRP levels were found to be more in females (37.8%) compared to males (19%) (*P* < 0.05) [16]. This variation has been also explained by the presence of immunologic differences between boys and girls [16].

In the current study, the median age of children with acute bronchiolitis was 3.7 (IQR, 1.27-12.3) months. Yet, other studies showed older age groups ranging from 5 months to 5 years [5, 8, 14, 15]. Patients with high CRP levels in the present study were older in age at presentation compared to those with normal levels (*P* < 0.0001). In an adult study published by Jeon et al., CRP levels were found to be increasing with age in patients with viral upper respiratory tract infections (*P* < 0.001) [5]. Yet, Resch et al. reported that CRP values were not influenced by the age of the infants at presentation (≤ three months or above) [13].

In the present study, median length of hospital stay was 5 (IQR, 3-8) days which was comparable to what was reported by Nagayama et al. (4 days, IQR 3-6.3) [16]. Length of hospital stay increases by the presence of bacterial coinfection which can lead to high CRP levels [1, 5, 6]. However, most of the reviewed studies did not report the length of stay of their patients [7, 10, 14, 15, 17].

The most common clinical presentations of patients with acute bronchiolitis in this study were cough (77.3%) and fever (74.7%), which is ingoing with the findings of several other studies [14–17]. Nonetheless, cough was more frequent in Lamarão et al. and Sawatzky et al. studies (97.9% and 93.3%, respectively); but the fever was of less frequency (72.4% and 51.7%, respectively) [14, 17]. In the present study, fever and cough were also found to be associated with high CRP levels (*P* < 0.0001 and *P* = 0.002, respectively). Do et al. study showed that fever is significantly associated with bacterial coinfection in children with RSV pneumonia (*P* < 0.01) [15]. However, on reviewing the literature, no previous study showed the relationship between frequency of fever and cough with the elevated levels of CRP in patients with acute bronchiolitis.

For the laboratory investigations, the current study had a median WBC count of 9.6 g/dL, which was similar to what was reported by Do et al. (9.7 g/dL) [15]. Mean WBC count in our study was higher in children with high CRP compared to those with normal levels, but this was not statistically significant. Similarly, Fares et al. found that WBC count was not predictive for bacterial coinfection in children with bronchiolitis [1]. Nonetheless, majority of children with viral infections have low WBC counts [7]. Moreover, WBC count did not differ between RSV-positive and RSV-negative infants in Resch et al.'s study [13].

In this study, patients with high CRP had lower hemoglobin levels compared to those with normal CRP levels (*P* < 0.0001). Papoff et al.'s study showed a trend of lower hemoglobin level in infants with severe bronchiolitis compared to those with mild-moderate disease which was in contradictory to their CRP levels [10].

RSV is the most common virus causing acute bronchiolitis [1]. In the present study, RSV infection was responsible for 36.7% of children presented with acute bronchiolitis. This percentage is higher than figures reported by Harbi et al. and Lamarão et al.'s studies (23.1% and 7.5%, respectively) [9, 14]. However, higher prevalence of RSV infections, ranging from 63.8% up to 84%, was found in other studies [4, 10, 15, 17]. These differences in the prevalence could be attributed to the seasonal variations of RSV infection and the time of study conduction. RSV infection can differ widely across latitudes and meteorological conditions [2]. It also peaks in the months of winter which can differ according to the geographical regions [1]. Despite that there was no significant difference between RSV-positive and RSV-negative patients in terms of the percentage of patients with high CRP levels, the mean CRP level was found to be significantly lower in RSV-positive (21.5 ± 27.7 mg/L) compared to RSV-negative patients (31.3 ± 44.3 mg/L) in this study (*P* = 0.042). Peltola et al.'s study showed that most children with viral infections has low CRP levels including those with RSV [7]. This finding might be attributed to the presence of a higher percentage of bacterial coinfections in the RSV-negative patients which might not be detected by blood, urine, or CSF cultures. However, Resch et al. found that CRP levels did not differ between RSV-positive and RSV-negative infants [13].

Patients with acute severe bronchiolitis who needed to be admitted to the PICU are usually sicker, may require mechanical ventilation, or have an associated bacterial coinfection. In contrary, those managed in general pediatric wards usually have a milder disease. Seriously ill infants with extensive consolidation or atelectasis had significantly higher CRP levels in Papoff et al.'s study (*P* = 0.04) [10]. Moreover, CRP values had a statistically significant relation with PICU admissions (*P* = 0.008) in Costa et al.'s study which hypothesized that CRP levels might serve as indirect markers of disease severity [18]. Accordingly, patients admitted to the PICU tend to have higher CRP levels compared to those not. Despite that the mean CRP levels in the present study were higher in patients admitted to the PICU compared to those not, this difference was not statistically significant. This study also showed no significant differences between patients with high CRP levels and those with normal levels in terms of complications and mortality rate. Similar to our study, Fares et al. and Resch et al.'s studies showed that acute bronchiolitis severity was not influenced by the CRP levels [1, 13].

The use of antibiotics in children with acute bronchiolitis in this study is considered high (78.1%), keeping in mind the low percentage of confirmed bacterial infections (17.6%). This is similar to the percentage reported by Alejandre et al. (79.9%) in pediatric patients with severe bronchiolitis [19]. Unnecessary use of antibiotics is common in children with acute bronchiolitis due to the challenging differential diagnosis between invasive bacterial infections and isolated viral infections [19]. Desmarest et al.'s study found an association between the antibiotic's prescription and high CRP levels [12]. Yet, these high levels could not predict the presence of alveolar condensation on the chest X-ray [12]. High CRP levels might mislead the physicians and force them to unnecessary use of antibiotics in a viral induced disease. For this reason, the decision of starting antibiotics in cases of acute bronchiolitis should be taken based on more rigorous evidence such as positive tracheal aspirate, blood, urine, or cerebrospinal fluid cultures rather than high CRP alone.

## 5. Study Limitations

This study was limited by missing some demographic and laboratory data. This can be justified by the retrospective nature of the study. Another limitation is the scarce number of published studies tackling the association between acute bronchiolitis and CRP levels which made the comparison between the results of our study with those of other studies difficult. Despite these limitations, the findings of this study are very important, being the first study to tackle this issue in Bahrain. Furthermore, although this is not an epidemiological study, we analyzed the IgG and IgM serology of some patients with available data. Serological tests can confirm RSV infection and help detect other causative organisms of acute bronchiolitis in RSV-negative patients. They are also important to be sure that RSV or other virus infection is of acute type and not because of previous infections or immunizations. This point can be considered as a strength for this study.

## 6. Conclusion

This study showed that most patients with acute bronchiolitis had high rate of elevated CRP values that did not correlate with the rate of bacterial coinfection. Children with high CRP levels were older at presentation, presented with more fever and cough, and had a lower hemoglobin level despite that those factors were previously reported to be associated with the disease severity and bacterial coinfection. This study also showed a high overall rate of antibiotic prescriptions in a mostly viral disease. Further studies to figure the critical CRP cut-off that might be of highly suspicious for bacterial infection and to build a clinical management algorithm to minimize the unnecessary use of antibiotics in children with acute bronchiolitis are needed.

## Figures and Tables

**Table 1:** Demographic data of children with acute bronchiolitis (*n* = 299).

Demographic data		No. of patients (%)
Sex	Male	132 (57.6)
	Female	97 (42.4)
Nationality	Bahraini	168 (73.4)
	Non-Bahraini	61 (26.6)
Gestational age (*n* = 188)	Term	153 (81.4)
	Preterm	35 (18.6)
Age at presentation (mon), median (IQR)		3.7 (1.27-12.33)
Current age (y), median (IQR)		1.37 (1.14-2.1)
Length of stay (d), median (IQR)		5.0 (3.0-8.0)

Values are presented as number (%) for categorical variables and median (interquartile range) for continuous variables. IQR: interquartile range.

**Table 2:** Blood investigations for 229 children with acute bronchiolitis.

Investigation	Mean	SD	Median	Minimum	Maximum	Normal range
White blood cells count (×10^6^/*μ*L)	11.4	8.6	9.6	0.8	111.4	3.6-9.6
Hemoglobin (g/dL)	11.3	2.2	10.9	5.7	20.0	12-14.5
Platelet's count (×10^6^/*μ*L)	418.5	176.4	393.0	14.5	971.0	150-400
C-reactive protein (mg/L)	27.5	39.0	10.4	0.1	297.0	0-3

SD: standard deviation.

**Table 3:** Comparison between C-reactive protein positive and negative patients.

Variable		C-reactive protein level (*n* = 299)		*P* value^∗^
		High, 167 (72.9)	Normal, 62 (27.1)	
Sex	Male	99 (59.3)	33 (53.3)	0.453
	Female	68 (40.7)	29 (46.8)
Nationality	Bahraini	128 (76.6)	40 (64.5)	0.092
	Non-Bahraini	39 (23.4)	22 (35.5)	
Gestational age (*n* = 188)	Term	109 (81.3)	44 (81.5)	1.000
	Preterm	25 (18.7)	10 (18.5)	
Age at presentation (mon), mean ± SD		11.76 ± 13.91	6.26 ± 17.60	<0.0001
Age at the time of study (mon), mean ± SD		32.22 ± 14.20	27.07 ± 17.44	<0.0001
Length of hospital stay (d), mean ± SD		10 ± 39	12 ± 69	0.253
History of fever		138 (82.6)	33 (53.2)	<0.0001
History of cough		138 (82.6)	39 (62.9)	0.002
White blood cells count (×10^6^/*μ*L), mean ± SD		11.92 ± 9.65	9.95 ± 4.78	0.131
Hemoglobin (g/dL), mean ± SD		10.9 ± 1.8	12.5 ± 2.7	<0.0001
Platelet's count (×10^6^/*μ*L), mean ± SD		417.3 ± 175.5	421.6 ± 180.1	0.910
Positive blood culture (*n* = 177)		13 (10.2)	4.0 (8.2)	0.783
Positive urine culture (*n* = 113)		9.0 (11.8)	4.0 (10.8)	1.000
Positive cerebrospinal fluid culture (*n* = 29)		1.0 (5.3)	0.0 (0.0)	1.000
Positive chest X ray (*n* = 228)		118 (71.1)	42 (67.7)	0.629
Positive RSV test (*n* = 226)		64 (28.3)	20 (8.8)	0.360
Antibiotic use (*n* = 225)		136 (81.4)	43 (69.4)	0.062
Complications		19 (11.4)	7.0 (11.3)	1.000
Admission to intensive care unit (*n* = 227)		9.0 (5.4)	4.0 (6.6)	0.751
Mortality (*n* = 228)		3.0 (1.8)	1.0 (1.6)	1.000

Values are presented as number (%) or mean ± SD. ^∗^Fisher's exact test was used for categorical variables, while Mann–Whitney *U* test was used for continuous variables. SD: standard deviation; RSV: respiratory syncytial virus.

**Table 4:** Binary logistic regression analysis of selected predictors of high C-reactive protein in 229 patients with acute bronchiolitis.

Variables	Adjusted odds ratio	95% CI	*P* value
Age at presentation (m)	0.883	0.764 to 1.021	0.094
Age at the time of study (m)	1.114	0.967 to 1.284	0.133
History of fever	2.492	1.190 to 5.222	0.016
History of cough	1.398	0.642 to 3.049	0.398
Hemoglobin (g/dL)	0.002	1.100 to 1.515	0.002

CI: confidence interval.

## Data Availability

The datasets generated during and/or analyzed during the current study are available from the corresponding author on reasonable request.

## References

[1] Fares M., Mourad S., Rajab M., Rifai N. (2011). The use of C-reactive protein in predicting bacterial co-infection in children with bronchiolitis. *North American Journal of Medical Sciences*.

[2] Piedimonte G., Perez M. K. (2014). Respiratory syncytial virus infection and bronchiolitis. *Pediatrics in Review*.

[3] Park H. W., Lee B. S., Kim A. R. (2012). Epidemiology of respiratory syncytial virus infection in infants born at less than thirty-five weeks of gestational age. *Pediatric Infectious Disease*.

[4] Kenneth M., Berman R. E., Kliegman R. M. (2000). Respiratory syncytial virus. *Nelson Textbook of Pediatrics*.

[5] Jeon J. S., Rheem I., Kim J. K. (2017). C-reactive protein and respiratory viral infection. *Korean Journal of Clinical Laboratory Science*.

[6] Alejandre C., Guitart C., Balaguer M. (2021). Use of procalcitonin and C-reactive protein in the diagnosis of bacterial infection in infants with severe bronchiolitis. *European Journal of Pediatrics*.

[7] Peltola V., Mertsola J., Ruuskanen O. (2006). Comparison of total white blood cell count and serum C-reactive protein levels in confirmed bacterial and viral infections. *The Journal of Pediatrics*.

[8] Higdon M. M., Le T., O’Brien K. L. (2017). Association of C-reactive protein with bacterial and respiratory syncytial virus–associated pneumonia among children aged <5 years in the PERCH study. *Clinical Infectious Diseases*.

[9] Al Harbi S., Kobeisy S. A. N., Hossain O. M. A., Faruqi K. M. A., Sayed B. Y. (2020). Respiratory pathogens in pediatric patients in Saudi Arabia: seasonal variation and epidemiological distribution. *Journal of Biomedical Science*.

[10] Papoff P., Moretti C., Cangiano G. (2011). Incidence and predisposing factors for severe disease in previously healthy term infants experiencing their first episode of bronchiolitis. *Acta Paediatrica*.

[11] Silver A. H., Nazif J. M. (2019). Bronchiolitis. *Pediatrics in Review*.

[12] Desmarest M., Aupiais C., Le Gal J. (2017). Value of procalcitonin for infants with bronchiolitis in an emergency department. *Archives of Pediatrics*.

[13] Resch B., Gusenleitner W., Müller W. (2003). Procalcitonin, interleukin-6, C-reactive protein and leukocyte counts in infants with bronchiolitis. *The Pediatric Infectious Disease Journal*.

[14] Lamarão L. M., Ramos F. L., Mello W. A. (2012). Prevalence and clinical features of respiratory syncytial virus in children hospitalized for community-acquired pneumonia in northern Brazil. *BMC Infectious Diseases*.

[15] Do Q., Dao T. M., Nguyen T. N., Tran Q. A., Nguyen H. T., Ngo T. T. (2020). Procalcitonin identifies bacterial coinfections in Vietnamese children with severe respiratory syncytial virus pneumonia. *BioMed Research International*.

[16] Nagayama Y., Tsubaki T., Nakayama S. (2006). Gender analysis in acute bronchiolitis due to respiratory syncytial virus. *Pediatric Allergy and Immunology*.

[17] Sawatzky J., Soo J., Conroy A. L. (2019). Biomarkers of systemic inflammation in Ugandan infants and children hospitalized with respiratory syncytial virus infection. *The Pediatric Infectious Disease Journal*.

[18] Costa S., Rocha R., Tavares M., Bonito-Vitor A., Guedes-Vaz L. (2009). C reactive protein and disease severity in bronchiolitis. *Revista Portuguesa de Pneumologia*.

[19] Alejandre C., Balaguer M., Guitart C. (2020). Procalcitonin-guided protocol decreased the antibiotic use in paediatric patients with severe bronchiolitis. *Acta Paediatrica*.

